# Supplementary material for: The interactome of intact mitochondria by cross-linking mass spectrometry provides evidence for coexisting respiratory supercomplexes
Source: Mol Cell Proteomics. 2017 Dec 8;17(2):216–32. doi: 10.1074/mcp.RA117.000470 (PMC5795388; doi:10.1074/mcp.RA117.000470)
Supplement: Supplemental Data [file supp_RA117.000470_133922_0_supp_23978_fzffwf.zip › spectra_annotation/mito_spectra_annotation/366-1-1-1-1-1.pdf]

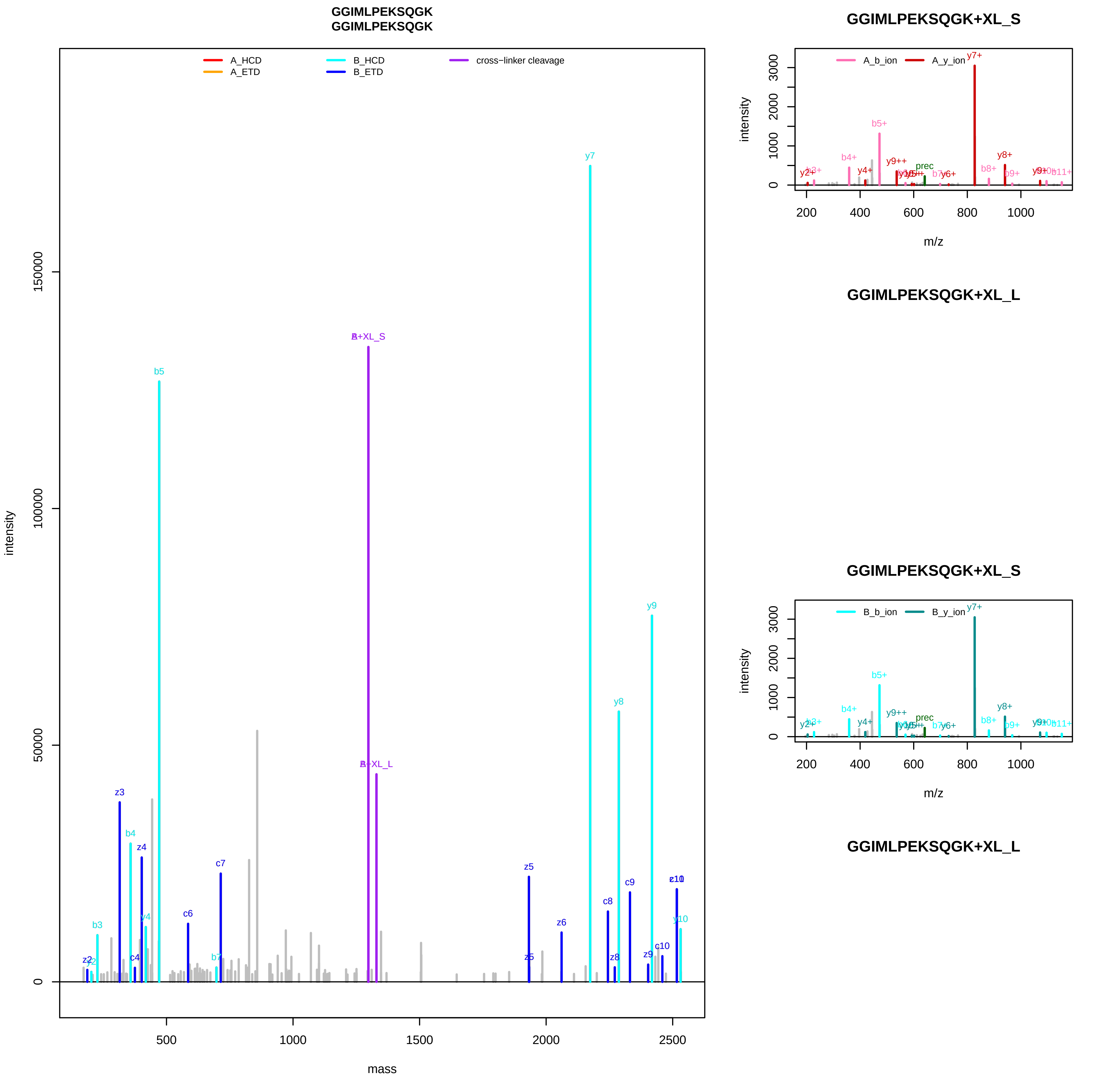
GGIMLPEKSQGK
GGIMLPEKSQGK
A_HCD
A_ETD
B_HCD
B_ETD
cross−linker cleavage
intensity
mass
0
50000
100000
150000
500
1000
1500
2000
2500
b3
z3
b4
z4
c4
y4
b5
c6
b7
c7
z5
z6
y7
c8
z8
y8
c9
z9
y9
c10
y10
GGIMLPEKSQGK+XL_S
A_b_ion
A_y_ion
intensity
m/z
prec
GGIMLPEKSQGK+XL_L
GGIMLPEKSQGK+XL_S
B_b_ion
B_y_ion
intensity
m/z
GGIMLPEKSQGK+XL_L
